# Regulation of 3D Organization and Its Role in Cancer Biology

**DOI:** 10.3389/fcell.2022.879465

**Published:** 2022-06-08

**Authors:** Anghui Peng, Wang Peng, Ruiqi Wang, Hao Zhao, Xinyang Yu, Yihao Sun

**Affiliations:** ^1^ Zhuhai Interventional Medical Center, Zhuhai Precision Medical Center, Zhuhai People’s Hospital, Zhuhai Hospital Affiliated with Jinan University, Jinan University, Zhuhai, China; ^2^ Guangdong Provincial Key Laboratory of Tumor Interventional Diagnosis and Treatment, Zhuhai Institute of Translational Medicine, Zhuhai People’s Hospital Affiliated with Jinan University, Jinan University, Zhuhai, China; ^3^ Department of Oncology, Liuzhou People’s Hospital, Liuzhou, China; ^4^ Department of Pharmacy, Zhuhai People’s Hospital, Zhuhai Hospital Affiliated with Jinan University, Jinan University, Zhuhai, China; ^5^ The First College of Clinical Medical Science, China Three Gorges University, Yichang, China

**Keywords:** chromatin, spatial structure, cancer, super-enhancer, oncogene

## Abstract

Three-dimensional (3D) genomics is the frontier field in the post-genomics era, its foremost content is the relationship between chromatin spatial conformation and regulation of gene transcription. Cancer biology is a complex system resulting from genetic alterations in key tumor oncogenes and suppressor genes for cell proliferation, DNA replication, cell differentiation, and homeostatic functions. Although scientific research in recent decades has revealed how the genome sequence is mutated in many cancers, high-order chromosomal structures involved in the development and fate of cancer cells represent a crucial but rarely explored aspect of cancer genomics. Hence, dissection of the 3D genome conformation of cancer helps understand the unique epigenetic patterns and gene regulation processes that distinguish cancer biology from normal physiological states. In recent years, research in tumor 3D genomics has grown quickly. With the rapid progress of 3D genomics technology, we can now better determine the relationship between cancer pathogenesis and the chromatin structure of cancer cells. It is becoming increasingly explicit that changes in 3D chromatin structure play a vital role in controlling oncogene transcription. This review focuses on the relationships between tumor gene expression regulation, tumor 3D chromatin structure, and cancer phenotypic plasticity. Furthermore, based on the functional consequences of spatial disorganization in the cancer genome, we look forward to the clinical application prospects of 3D genomic biomarkers.

## Introduction

In 1885, Carl Rabl first introduced the concept of the regional organization of interphase chromosomes inside the animal nucleus. He predicted the orientation of interphase chromosomes and the occupation of different regions throughout interphase, providing insights into the chromosomal arrangement in the nucleus (Cremer and Cremer, 2006b). In 1909, Theodor Boveri put forward the term chromosomal territories (CTs) and noted that each chromosome visible in the cell nucleus occupies a distinct part of the nuclear space (Cremer and Cremer, 2006a). At the end of the 20th century, Cremer and colleagues confirmed the presence of CTs using electro fluorescence imaging (Cremer et al., 1982), revealing the dynamic architecture of chromatin and disseminating potential implications in the functional compartmentalization of the nucleus. The 2-m-long DNA in eukaryotic cells is highly compacted into the nucleus in the form of chromatin, with nucleosomes as basic subunits that organize DNA and histones into a compact chromatin state (Handoko et al., 2011). Epigenetic modifications of histones affect the affinity of DNA-bound proteins, leading to changes in chromatin configuration (Zhu and Li, 2016). At higher levels, euchromatin and heterochromatin regions are often spatially separated in the same CT (Hildebrand and Dekker, 2020). Pioneering studies have confirmed that chromosome location, chromatin structure, and transcriptional regulation are closely intertwined (Rennie et al., 2018).

The human genome comprises more than 2,000 topologically associated domains (TADs), covering more than 90% of the genome (Gorkin et al., 2014). TAD boundaries act as effective insulators that distinguish transcriptional regulatory activities from potential targets, thereby increasing the frequency of chromosomal contacts (Handoko et al., 2011; Bonev et al., 2017). One of the key factors in the formation of the TAD boundary is the interaction between the zinc finger transcription factor CTCF and the multi-subunit protein complex cohesion (Szabo et al., 2019). TAD acts as a combination of self-interacting domains bound by multiple nested CTCFs (Rao et al., 2014). However, the mechanism by which CTCF isolates chromatin interactions between TADs has not been comprehensively elucidated. To date, two hypothetical models, the handcuff model, and the ring extrusion model have been proposed to explain it. On the one hand, in the handcuff model, CTCF spans TAD boundaries, and the two ends of TAD are connected by CTCF protein which recruits cohesion (Vietri Rudan and Hadjur, 2015; Dixon et al., 2016). On the other hand, the ring extrusion model proposes that the mammalian genome is divided into TADs in the megabase range on average, with a pair of tethered DNA-binding units sliding along the DNA in opposite directions to form DNA loops, with the DNA between the units extruding out (Dekker and Mirny, 2016). It can predict the binding specificity of the CTCF protein (Xi and Beer, 2021).

In addition to the enrichment of CTCF domains, the TAD boundary also contains a large number of DNA elements such as housekeeping genes, tRNAs, and short interspersed element (SINEs) retrotransposons (Lupianez et al., 2016). TAD organization divides chromatin compartments into type A (open domain, gene-rich) and type B (closed domain, gene-poor), which alternate along the chromosome and are approximately 5 Mb long (Dekker et al., 2013; Hildebrand and Dekker, 2020). A high-resolution multiple interactions map of the 4.5 Mb domain in the mouse X chromosome inactivation center showed that intra-TADs interactions were stronger than inter-TADs interactions (Nora et al., 2012). In general, TAD is highly conserved in different cell types, whereas compartments A and B, and gene expression patterns in open chromatin loci, are highly cell type- and tissue-specific (Thurman et al., 2012; Fortin and Hansen, 2015). Complex DNA topologies, including polymer loops, are frequently coupled with specific interaction kinetics of proteins and DNA molecules on target sequences (Zhang et al., 2006). Transcriptional regulation plays a critical role in lineage differentiation and cell fate determination in eukaryotes. This complex transcriptional system comprises a series of regulatory elements, such as enhancers and super-enhancers (SEs) that finely tune target gene expression (Wray et al., 2003; Prieto and Maeshima, 2019). Enhancers are short cis-regulatory elements, whereas SEs spanning dozens of kilobases are clusters of putative enhancers playing decisive roles in defining cellular identity (Pott and Lieb, 2015; Peng and Zhang, 2018). In human cells, most enhancers interact remotely with the promoters of target genes, whereas only a few enhancers regulate proximal promoters (Mora et al., 2016). Enhancers play an important role in the active establishment of chromatin loops. Because enhancers can be physically associated with the promoter of the target gene by 3D circularization or tracking, chromatin interactions are not always linearly proximal (Lettice et al., 2003; Montavon et al., 2011; Proudhon et al., 2015). The effects of long-range promoter-enhancer interactions appear to be mediated, in part, by loop formation. In other words, the loop structure enables the long-range regulation of target genes.

Chromatin structure alterations are a major cause of transcriptional dysregulation in various diseases, including cancer. The stable 3D chromatin state ensures precise gene expression by organizing regulatory elements and gene loci at close spatial distances, thereby ensuring the normal structure and function of the genome (Ma et al., 2019). The specific subsets of oncogenes expressed by each cell are directly related to gene regulation and transcriptional activity (Vicente-Duenas et al., 2013). The 3D genome structure of tumor cells is clearly distinguishable, and its TAD structure is smaller than that of normal cells (Taberlay et al., 2016). Tumorigenesis is often accompanied by a large number of mutations, and the mutated genes are high efficiently transcribed in broadly accessible chromatin regions. Transcribed regions are reassigned to greater spatial proximity, enabling genes to share regulatory elements and transcriptional factors (TFs) (Mourad et al., 2014). Alterations in the chromatin spatial structure of tumor cells promote the formation of different combinations of enhancers and oncogenes in the dynamic transcription process. Additionally, the causal relationship between heterochromatin dysfunction and increased genomic instability is a well-established mechanism underlying cancer progression. Given the significance of genome topology, an increasing number of unsolved issues are related to how it affects human cancer biology.

### Main Technologies of 3D Genomics

Over the past few decades, an increasing number of tools have been developed to study the physical organization and transcriptional regulation of genomes. Advanced techniques have made it possible to capture alterations in chromatin conformation during different developmental stages inside the nucleus. Gradually, more and more technologies aimed at 3D chromatin spatial detection have emerged. The major 3D genomics techniques are listed in Table 1.

**TABLE 1:** Main technologies of 3D genomics.

Technologies	Characteristics	Advantages	Limitation	Reference
3C	The interaction mode is one versus one	Precisely detects the interaction between two target regions	Low throughput; low resolution	Dekker et al. (2002)
4C	Reverse PCR; the interaction mode is one versus all	Detects the interactions between one target region with genome	Interaction data are prone to bias	Simonis et al. (2006)
5C	Multiple Primer design; the interaction mode is many versus many	Detects interactions among multiple regions	Low coverage and difficult-to-assess PCR redundancy	Dostie et al. (2006)
Hi-C	Interaction mode is all versus all	High-throughput detection of genome-wide interactions	High cost of sequencing; difficult to analyze because of the large amount of data	Lieberman-Aiden et al. (2009)
Capture-C	Target domain capture	Provide an unbiased, high-resolution map of cis interactions for hundreds of genes in a single experiment.	Sampling is limited to a defined domain of chromatin	Hughes et al. (2014)
3D FISH	DNA imaging scheme in single cells	Highly multiplexed detection of a genomic region of interest	Harsh treatments are required to prepare the chromatin for the FISH probes	Solovei et al. (2002)
DNase-HiC	Endonuclease DNase I replaces the restriction endonuclease	Higher effective resolution than traditional Hi-C libraries	DNase exhibits sequence bias at cleavage sites with low GC content	Ramani et al. (2016)
Micro-C	Micrococcal nuclease replaces the restriction endonuclease restriction enzymes	Able to access shorter-range interactions at higher resolution	Cannot capture long-range interactions	de Souza, (2015)
ChIP-seq	Genome-wide profiling of DNA-binding proteins, histone modifications, or nucleosomes	High resolution, low noise, great coverage, and decreased cost of sequencing	Difficulty in analyzing data owing to bias	Park, (2009)
ATAC-seq	DNA accessibility with hyperactive Tn5 transposase	Fast and sensitive detection for genome-wide chromatin accessibility	Difficult to achieve ideally cut fragments	Buenrostro et al. (2015)
ChIA-PET	Protein-centric chromatin conformation method	High-throughput detection of protein-mediated genome-wide interactions	Difficult to obtain specific antibodies for protein detection	Li et al. (2017)
HiChIP	Protein-centric chromatin conformation method	More efficient and lower input requirement than ChIA-PET; multi-scale genome architecture with greater signal to the background than *in situ* Hi-C	Biased signal owing to the enrichment of target binding sites	Mumbach et al. (2016)

Dekker et al. developed chromatin conformation capture (3C), which, along with its derived technologies, such as 4C, 5C, HiC, and ChIA-PET, has allowed genome-scale detection of long-range interactions between specific sites of chromatin in candidate regions (Dekker et al., 2002; Sati and Cavalli, 2017), revealing the hierarchical structure of chromosomes and providing information on the organization and interaction of chromatin in different cell types. Chromosome conformation capture-on-chip (4C) can generate a genome-wide interaction map of multiple sites with a bait sequence (Simonis et al., 2006). Chromosome conformation capture carbon copy (5C) technology allows for the chromatin interactions of a large number of genes by drawing an interaction map between multiple loci (Dostie et al., 2006). High-throughput chromosome conformation capture (Hi-C) technology, which can capture all chromatin interactions in the whole genome, is currently a robust tool over mass capture technologies to identify chromatin loops and describe TAD compartment conditions (Lieberman-Aiden et al., 2009).

Although HiC reveals TADs as conserved features of chromatin organization, it is limited to the observation of thousands of cells and the reliance on restriction enzymes for fragmentation. Some techniques can make up for these limitations. FISH on 3D-preserved nuclei (3D-FISH) in combination with 3D-microscopy and image reconstruction provides detailed information on the chromatin architecture by visualizing individual chromosomes at the interphase stage, thus providing direct evidence for CTs in the nucleus at the single-cell level (Solovei et al., 2002; Cremer and Cremer, 2010). DNase Hi-C and Micro-C use DNase I and micrococcal nuclease (MNase), respectively, instead of digesting cross-linked genomes, generating mononucleosomes, and inferring genome structure maps at single-nucleosome resolution (de Souza, 2015; Ramani et al., 2016). The relentless development of 3D genomic techniques led to cutting-edge technologies. Capture-C yields hundreds of fold fragment enrichment, significantly improving the detection efficiency of local interactions in target chromatin regions (Hughes et al., 2014). Chromatin immunoprecipitation (ChIP) technology is an effective tool for investigating TFs and histone modifications (Park, 2009). The assay for transposase-accessible chromatin (ATAC) technique reveals the chromatin state of most noncoding functional elements in the whole genome (Buenrostro et al., 2015). In addition, chromatin interaction analysis by paired-end tag sequencing (ChIA-PET) (Li et al., 2017) and HiChIP (Mumbach et al., 2016) can comprehensively capture specific protein-mediated interactions in the whole genome. Combined with high-throughput sequencing, these technologies provide a way to understand how eukaryotic genomes fold and organize inside the nucleus.

**FIGURE 1:**
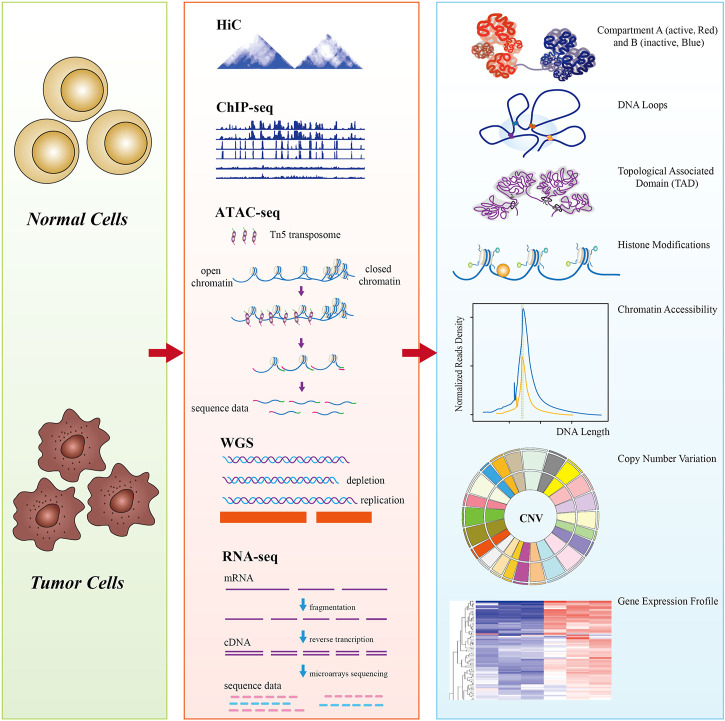
A schematic diagram of multi-omics analysis between normal cells (control) and tumor cells. Hi-C data showed that tumor chromosome territories could be partitioned into A (active, red) and B (inactive, blue) compartments, chromatin is folded into topologically associating domains (TADs) (100–1,000 kb), and enhancer–promoter loops (10–500 kb); ChIP-seq revealed tumor genome-wide epigenetic changes, such as histone modifications; ATAC-seq detects tumor genomic chromatin accessibility using Tn5 transposase-specific recognition cleavage of open chromatin; whole-genome sequencing (WGS) detects tumor chromatin structural variations, including copy number variations (CNVs); genome-wide detection of tumor-specific genes by RNA-seq. Multi-omics reveals the hierarchical structures of 3D genome organization, transcription regulation, and structure variation mechanisms of the whole tumor genome at the genetic, epigenetic, and RNA levels.

From the perspective of the multi-omics level of DNA mutation, epigenetic alterations, histone modification, 3D conformation, and transcriptional regulation, Hi-C is generally combined with one or more additional techniques (whole-genome sequencing (WGS), ChIP-seq, ATAC-seq, and RNA-seq) to investigate the transcriptional regulation and pathogenetic mechanisms of cancers (Figure 1). With the rapid development of single-cell technology, multi-omics have opened up new avenues for revealing the tumor cell pathogenesis and underlying regulatory mechanisms.

### Transcriptional Dysregulation Participates in Oncogenesis

The smooth operation of gene expression patterns plays a fundamental role in the finely-tuned regulation of gene expression. Transcriptional dysregulation triggers carcinogenesis, including abnormal cellular processes, such as hyperproliferation, immortality, metastasis, and immune escape (Liu et al., 2018; Gupta et al., 2020). Cis-regulatory elements control tissue-specific gene expression underlying tumor cell development, determining cell identity and cell fate (Huang et al., 2021). SEs can activate oncogene expression, irrespective of the distance or orientation to their transcription start sites (Tang et al., 2020). The SE-associated transcription program is key to revealing the mechanism of tumorigenesis (Zhang T. et al., 2020). In a wide array of cancer types, intensive transcription of oncogenes in cancer cells is often promoted by SEs (Sengupta and George, 2017). *MYC* is a classical SE-associated oncogene involved in global gene transcription amplification. The *MYC* members of human oncogenes include *c-MYC*, *MYCL*, and *MYCN*. *MYCN* protein is an oncogenic driver that functions in transcriptional programs similar to those of *MYC* (Zanotti et al., 2021). Cutting-edge research has pointed out that the association of SEs with multiple oncogenes is acquired during hepatocarcinogenesis, and the increase in SEs at *MYC* and *MYCN* was observed in hepatocellular carcinoma (HCC) cells (Tsang et al., 2019). In osteosarcoma and multiple myeloma (MM), most SE-amplified genes are bound by *MY*C (Loven et al., 2013; Chen et al., 2018). Xiang et al. found that a super-enhancer of approximately 150 kb located 515 kb upstream of *MYC* forms a chromatin loop with *MYC* in human colorectal cancer (Xiang et al., 2014). *c-MYC* is abnormally highly expressed in the process of B cell carcinogenesis owing to the chromatin space remodeling (Jiang S. et al., 2017). The inhibition of transcriptional cyclin-dependent kinases (CDKs) leads to global repression of *MYCN*-dependent transcriptional amplification and sustained growth of neuroblastoma cells. In line with this, the upregulation of the active transcriptional program in neuroblastoma cells is promoted by the development of SEs (Chipumuro et al., 2014). Yuan et al. integrated RNA-seq and ChIP-seq to explore SE-mediated transcriptional dysregulation in nasopharyngeal carcinoma (NPC) cells by screening 19 SE-associated candidate genes (Yuan et al., 2017). They validated that five genes (*BCAR1*, *F3*, *LDLR*, *TBC1D2*, and *TP53TG1*) sustain the cell survival and promote proliferation of NPC. DNA-binding motif analysis has shown that *ETS2* is a potential SE-promoting TF during NPC tumorigenesis (Yuan et al., 2017). *RUNX1* and *DNAJB1*, identified as SE-associated oncogenes in esophageal squamous cell carcinoma (OSCC), significantly promote OSCC cell proliferation (Jiang Y. Y. et al., 2017). In prostate cancer cell lines and tissues, two enhancers located 63 kb upstream and 48 kb downstream of the *PTBP3* region were identified to specifically loop to the *PTBP3* promoter (Kubiak et al., 2019). Overall, cell type-specific gene transcriptional dysregulation is the hallmark of malignancies and is primarily underpinned by alterations in SEs. The dependence on SE-driven transcription in cancer biology greatly benefits tumorigenesis. Aberrant cell growth and proliferation prompted by dysregulated transcriptional progression renders cancer highly invasive and unconducive to clinical therapy.

### Alterations of 3D Genome Architecture in Cancers

In many tumor types, decompressed heterochromatin leads to decreased chromosomal stability, DNA damage, fragmented DNA folding, and activated transcription, eventually triggering the malignant transformation in the early stage of carcinogenesis (Xu et al., 2020). Transcriptional differences increase gene expression in the transition domain of type B to type A compartment, promoting interactions in type A compartments on chr16-22 in breast cancer (Barutcu et al., 2015). Significant differences in the stereotypical folding of each chromosome which boosts gene expression in B-type to A-type compartment conversion regions were observed in genome-wide chromatin conformation between normal epithelial cells and breast cancer cells (Barutcu et al., 2015). In T cell acute lymphoblastic leukemia (T-ALL), the loss of boundary sites of TADs, which may support the gene regulation theory by promoting enhancer promoter interactions and isolating different regulatory units, can activate oncogenes insulated neighborhoods (Hnisz et al., 2016). Li et al. found that the alteration of CTCF binding, which disrupts the robustness of the TAD boundary, interferes with the oncogenic transcription program of the *TAL1* gene, dramatically altering leukemogenic processes. The polarity and organization of the TAD boundary depend on the CTCF orientation (Li et al., 2020). Kloetgen et al. integrated Hi-C, RNA-seq, and CTCF ChIP-seq technologies, revealing that TAD boundary disruption is associated with increased enhancer promoter interactions and chromatin accessibility (Kloetgen et al., 2020). Zhou et al. uncovered 24 dynamic patterns characterizing 3D genome recompartmentalization accompanied by lower CTCF binding at the TAD boundary in estradiol (E2)-induced breast cancer cells (Zhou et al., 2019). The conformation of the 3D chromatin genome is a deeper layer of inter-tumor heterogeneity. In glioblastoma, specific boundary loss causes the enhancer to interact abnormally with the oncogene *PDGFRA* (Lettice et al., 2003). The immune-related gene *CD276,* which co-expressed with stem cell genes, displays increased accessibility in glioblastoma stem cells to achieve a shared 3D genome state that triggers self-renewal. It is thought that genome instability destroys the normal transcription program (Johnston et al., 2019). Collectively, high-resolution 3D tumor genome maps provide global insights for evaluating cancer transcription programs, genome stability, and compartment conversions. The integration of information on loops, territories, and compartment construction contributes to a comprehensive understanding of tumor genome organization and etiology.

### Tumor Structure Variation and TAD Boundary

In most cancers, structural variants promote oncogenesis through a variety of mechanisms, including the genome with complete or partial chromosomal gain and loss. A comprehensive understanding of the entire cancer system is required to dissect the interplay between higher-order chromatin structures and somatic mutations (Harbers et al., 2021). Multiple structural and numerical chromosomal aberrations lead to profound changes in the structure and function of the genome, including translocations, insertions, point mutations, copy number variations (CNVs), and chromosomal aneuploidy (Teixeira and Heim, 2005). These variations are hallmarks of most cancer genomes. Cancer epigenetics and genetics may have complementary roles in this regard. A typical example is the Philadelphia chromosome (Ph) first discovered by Nowell and Hungerford and described as a typically short chromosome 22 recurring in tumor cells of patients with chronic myelogenous leukemia (CML) (Nowell et al., 1960).

Increasing evidence has demonstrated that chromosomal translocation coupled with the disruption of 3D genome organization plays a role in carcinogenesis. A study on carcinogenic translocation events suppressed by tyrosyl-DNA phosphodiesterase 2 (*TDP2*) found that the loss of non-homologous end joining (NHEJ) repair during transcription disrupts genome stability (Ramsden and Nussenzweig, 2021). The frequency of translocation selection is related to the spatial contact probability of interaction sites. In MM, CNV breakpoints overlap with the TAD boundaries. By integrating Hi-C, WGS, and RNA-seq data of MM cell lines, Wu and colleagues identified 56 inter-chromosomal translocations with multiple inter-chromosomal interactions. The intensity of the overall spatial interaction between chromosomes of MM cell lines is significantly higher than that of normal B cells, indicating that the 3D conformation of the cancer cell genome is affected by inter-chromosomal translocations during MM development (Wu et al., 2017). Another cause of tumor genome instability is double-strand breaks (DSBs) during gene transcription, possibly resulting in chromosomal translocation. Translocations are likely to occur at hotspots of DSBs in regions with extreme spatial proximity (Zhang et al., 2012). Furthermore, specific 3D FISH chromatin landscapes unveil gene activity-related changes containing spatial relationships of DNA-proteins and translocation in human cancers (Kocanova et al., 2018; Kulasinghe et al., 2020).

Tumor structure variations are involved in cancers, as they can affect TAD integrity, reorganize specific enhancer promoter interactions, and alter gene expression (Anania and Lupianez, 2020). Insulator proteins such as CTCF bind to the TAD boundary, preventing the interactions of genes and regulatory elements between different TADs (Kim et al., 2007). However, a recent study documented that TAD boundary destruction can alter the TAD structure and establish new TADs (Ulianov et al., 2016). New domains can also be established without destroying the TAD boundaries. For example, genomic rearrangement with breakpoints in TADs leads to their breakage and fusion, ultimately activating oncogenes and ultimately triggering tumorigenesis (Groschel et al., 2014; Northcott et al., 2014). Dixon et al. found extensive deletion of enhancers at the distal end of the region where the structural mutations occurred. Enhancers are located near genes that are frequently mutated in cancers (Dixon et al., 2018). Prostate cancer cells retain the ability to segment their genome into megabase-sized TAD regions and establish new smaller cancer-specific TADs, whose boundaries mostly appear in the CNV area (Taberlay et al., 2016). Although the genome of tumors typically has more TADs, their average TAD size is smaller than that of normal cells (Wu et al., 2017). Oncogene dysregulation can be caused by the loss or reduced activity of TAD boundaries. Gain-of-function mutations in IDH are characteristic of the main pathological and treatment prognostic categories of gliomas. Flavahan and colleagues found that CTCF binding sites are significantly reduced in IDH mutant gliomas, allowing a potent enhancer to aberrantly contact and activate *PDGFRA* expression (Flavahan et al., 2016). CTCF site depletion at the TAD boundary and variation in chromatin structure are found in the aberrant expression of pathogenicity-related genes in some cancers. A general genome-wide dysregulation of gene expression associated with TAD boundaries has been found in B cell precursor acute lymphoblastic leukemia (BCP ALL) in hyperdiploid children. Hyperdiploid ALL shows abnormal chromosome morphology, whereas low expression of CTCF and cohesin is observed in hyperdiploid ALL (Yang et al., 2019).

Overall, structural variation in the chromosomal aberration program of the cancer epigenome leads to chromatin remodeling and dysregulated gene expression, whose malignant mechanism is related to the destruction of TAD boundaries.

### Outlook of 3D Genomics in Tumor Diagnosis and Treatment

**FIGURE 2:**
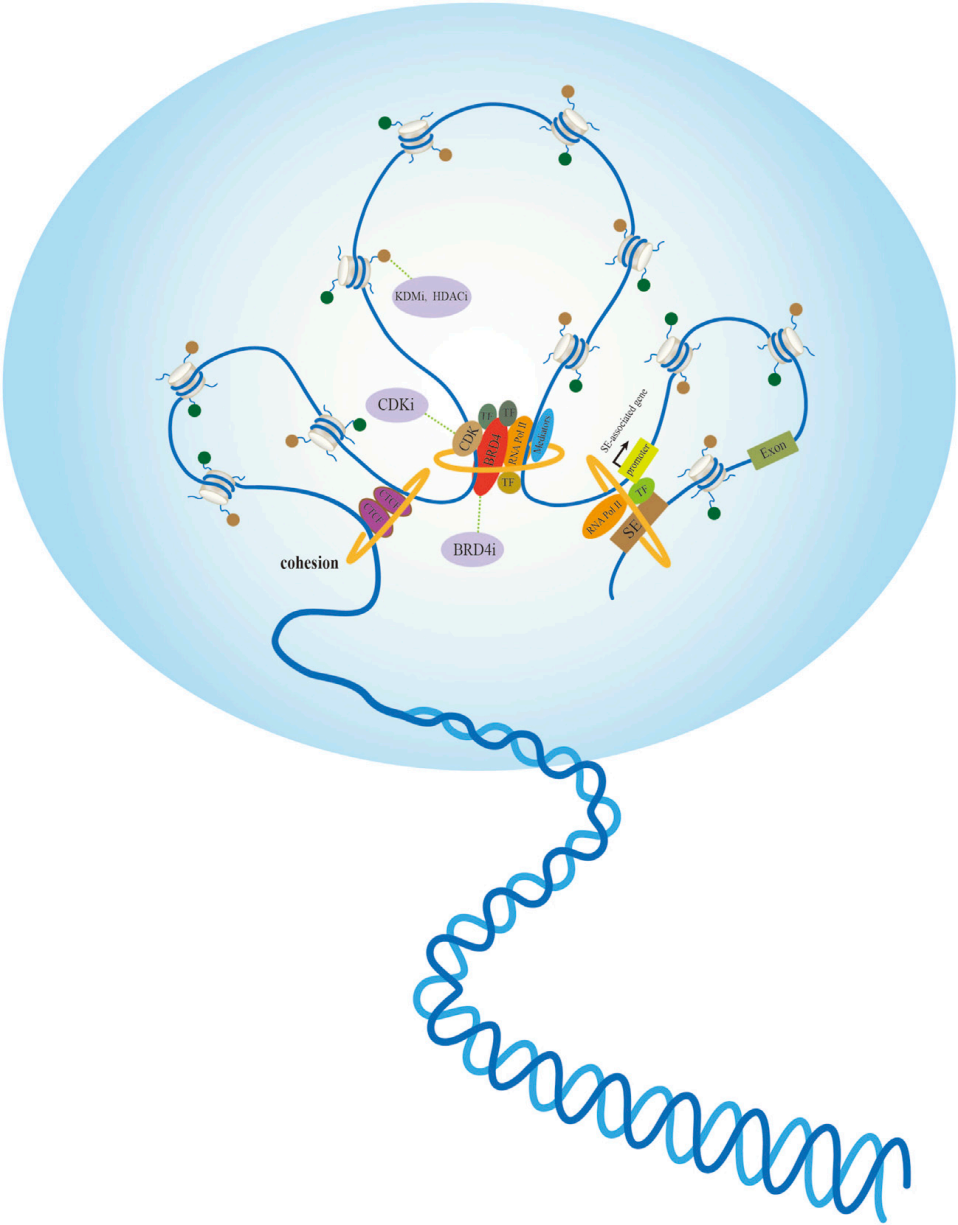
Active chromatin hubs of tumor nuclear morphology and potential anticancer targets. Left: The internal structure of chromatin loop formed by spatial contacts in CTCF binding sites. Middle: Multiple proteins containing transcription factors (TFs) recruit mediators and RNA polymerase II (RNA Pol II) participates in nuclear transcription *via* different mechanisms. Small-molecule inhibitors exert anticancer effects by targeting tumor-promoting proteins or histone modifications. Right: Spatial dimension of SE-associated gene regulation in a gene-specific manner, transcription factor (TFs) binding to super-enhancers (SE) facilitates interaction with promoters with large genomic distances.

Because cancer is characterized by morphological changes in the cell nucleus, exploring the chromatin structure in cancer is expected to help identify candidate biomarkers (Figure 2). High-throughput analysis of genome-wide histone modifications shows that in almost all cancer types, a group of genes have unique epigenetic characteristics that are closely related to different stages and different kinds of tumors. Currently, epigenetic markers are used as effective biomarkers in early clinical screening and the prediction of patient diagnosis and treatment response. Identifying specific histone signatures associated with each type of cancer enables not only a more accurate diagnosis and prognosis, but also lays the foundation for the design and evaluation of epigenetic agents (Audia and Campbell, 2016). The use of inhibitors of DNA methyltransferases and HDACs is clinically effective for several cancers. For instance, several KDMs in the family of histone lysine demethylases have been implicated in the development of various cancers, and are thus considered potential drug targets. KDM inhibitors have potential value for elucidating tumor cell function and tumor therapy (Hoffmann et al., 2012; McAllister et al., 2016). Moreover, HDAC8 knockdown initiates a similar differentiation program as selective small-molecule inhibitors in neuroblastoma cells (Oehme et al., 2009). Clinical implications of biological programs allow the design of HDAC8-selective small-molecule inhibitors for cancer cell suppression.

The mechanisms by which oncogenes control myriad cellular processes to induce tumorigenesis expose the fragility and difficulties in treatment. Transcriptional inhibitors are potential therapeutic agents for treating certain tumors. In *MYC*-dependent cancers, interference of chromatin-dependent signal transduction with RNA polymerase II (RNA Pol II) and inhibition of RNA Pol II transcription initiation and elongation are therapeutic principles in malignancies. CDK7, a member of a family of CDKs involved in regulating RNAPII initiation, pause, and elongation, preferentially binds to SE and activates SE-related gene expression (Larochelle et al., 2012). The selective targeting of mechanisms that promote the overall transcriptional amplification in tumor cells renders CDK7 inhibition an effective target for the treatment of cancers driven by specific oncogenes (Chipumuro et al., 2014). The blockade of CDK7 function is expected to suppress the expression of genes primed for transcription. For instance, CDK7 inhibitors commonly repress *MYC*, an oncogene overexpressed in 70% of human cancers. THZ1 is a small-molecule covalent inhibitor of CDK7 that blocks *MYC/MYCN* transcription in *MYC/MYCN*-amplified cells by irreversibly inhibiting CDK7. The unique SE landscape of *MYCN*-amplified cells also determines their sensitivity to THZ1, which protects normal cells from toxicity (Chipumuro et al., 2014). THZ1 also exerts a prominent anticancer effect on HCC and NPC (Yuan et al., 2017; Tsang et al., 2019). The bromodomain and extra-terminal domain (BET) family protein BRD4 has been recognized as a general regulator that couples the acetylation state of chromatin with Pol II elongation (Jang et al., 2005). Transcriptional dysregulation of BRD4 promotes the transcriptional activation of specific downstream targets that promote malignancies (Yang et al., 2005; Muhar et al., 2018). BRD4 is closely associated with tumorigenesis and has shown therapeutic potential in preclinical models (Shi and Vakoc, 2014; Wu et al., 2019). JQ1 is a small-molecule BRD4 inhibitor that targets the acetyllysine-recognition domain (bromodomain) of a putative coactivator involved in transcription initiation and elongation to repress *MYC* transcriptional function by the competitive displacement of chromatin-bound coactivators. Bromodomain inhibitors may be an ideal model system for agent mechanism and translational research on *MYC* pathway inhibitors (Delmore et al., 2011; Donati et al., 2018). Surprisingly, dihydroergotamine (DHE), an *NR4A*-induced drug, showed similar efficacy as JQ1 in inhibiting SE-dependent *MYC* transcription and AML growth in mouse xenografts (Call et al., 2020). It implies that DHE is a promising alternative therapeutic strategy for BET inhibitors in AML. These small-molecule inhibitors provide novel therapeutic strategies for specific malignant diseases. However, the limitations of poor prognosis and the emergence of drug resistance render their therapeutic effects unsatisfactory. Notably, combinatorial therapy with BRD4i and histone deacetylase inhibitors (HDACi) showed strong synergy in reducing tumor burden and inhibiting tumor progression (Mazur et al., 2015). Combining JQ1 and THZ1 in treating head and neck squamous cell carcinoma (HNSCC) effectively inhibited tumor growth and reduced toxicity and drug resistance, resulting in a better prognosis for patients (Zhang W. et al., 2020). A synergistic effect was also observed coupling BRD4 inhibitors (BRD4i) and CDK inhibitors (CDKi) in the treatment of medulloblastoma (Bandopadhayay et al., 2019).

As multiple cancer subtypes are rapidly emerging, epigenetic modulators of specific modifications and small-molecule inhibitors of tumor-promoting factors have become entailing hallmarks. The combined inhibition of these regulatory proteins is an alternative therapeutic strategy for cancer clinics. Whether epigenetic alterations and transcriptional regulations are the cause or the result of altered cellular states, they have potential value as biomarkers for disease diagnosis or as targets for therapeutic intervention. In the long run, systematic interrogation of cancer entities and pathologies of aberrant chromatin folding will uncover new vulnerabilities and novel therapeutic targets in personalized therapy.

## Conclusion

The integrity of the 3D hierarchical structure of chromosome entities throughout the life cycle of human cells is important for the proper deployment of cell-type-specific gene expression programs. Abnormalities in chromosomal integrity and structure, such as aberrant chromatin folding, compartment conversions, disruption of TAD boundaries, and rewiring of promoter-enhancer interactions generally lead to malignant transformation *via* dysregulated gene expression. The interplay between transcription and genome conformation is the driving force behind cell fate determination, and 3D genome structure plays a critical role in characterizing cancer, thus having profound clinical implications. With the deepening of research on the higher-order chromatin structure of tumor cells, we might gain a more comprehensive understanding of the pathophysiology of carcinogenesis, ultimately promoting the development of clinical cancer treatment.
